# The Effect of Intravenous Immunoglobulin Combined with Corticosteroid on the Progression of Stevens-Johnson Syndrome and Toxic Epidermal Necrolysis: A Meta-Analysis

**DOI:** 10.1371/journal.pone.0167120

**Published:** 2016-11-30

**Authors:** Liang-ping Ye, Cheng Zhang, Qi-xing Zhu

**Affiliations:** 1 Institute of Dermatology and Department of Dermatology at No.1 Hospital, Anhui Medical University, Hefei, Anhui, China; 2 Physical Examination Centre, The First Affiliated Hospital of Anhui Medical University, Hefei, Anhui, China; 3 Department of Occupational and Environmental Health, School of Public Health, Anhui Medical University, Hefei, Anhui, China; China Medical University, TAIWAN

## Abstract

**Background:**

Intravenous immunoglobulin (IVIG) treatment is commonly used to treat Stevens-Johnson syndrome (SJS) and toxic epidermal necrolysis (TEN) with controversial therapeutic effect.

**Methods:**

We conducted a comprehensive meta-analysis through combining the published eligible studies to evaluate the effectiveness of IVIG on SJS and TEN treatment.

**Results:**

A total of 26 studies were selected from public available databases. The combination of IVIG and corticosteroid markedly reduced the recovery time (by 1.63 days, 95% CI: 0.83–2.43, *P* < 0.001), compared with solo corticosteroid group. The favorable effects were greater in Asian (2.19, 95% CI: 1.41–2.97, *P* < 0.001), TEN (2.56, 95% CI: 0.35–4.77, *P* = 0.023) and high-dose IVIG treated individuals (1.78, 95% CI: 0.42–3.14, *P* = 0.010). The hospitalization length reduced by 3.19 days (95% CI: 0.08–6.30, *P* = 0.045), though the outcome was proven to be unstable. We found heterogeneities, which sources were probably regional factors. Besides, IVIG was inclined to decrease SJS/TEN mortality (SMR: 0.84, 95% CI: 0.66–1.08, *P* = 0.178). This impact was possibly more profound when patients were treated with high dose IVIG (SMR: 0.74, 95% CI: 0.50–1.08, *P* = 0.116), or when patients were diagnosed as TEN (SMR: 0.68, 95% CI: 0.45–1.01, *P* = 0.058).

**Conclusions:**

Our current meta-analysis suggests that IVIG combined with corticosteroid could reduce recovery time for SJS and TEN. This effect is greater among Asian patients. Whereas, its impact on reducing mortality is not significant.

## Introduction

Stevens-Johnson syndrome (SJS) and toxic epidermal necrolysis (TEN) are two severe types of drug hypersensitivity characterized by extensive epidermal separation from dermis and the degree of which is used to distinguish the clinical classification[1, 2]. SJS, SJS/TEN overlap and TEN refer to detachments on <10%, 10~30% and > 30% of the body surface area, respectively. Despite the characteristic epidermal detachment, a considerable proportion of patients may suffer from various acute complications. The most common sequelae is reported as ocular involvement which occurs in more than half of SJS/TEN patients [3]. SJS/TEN also results in long-term morbidities involving various organs, which is well documented in the latest review[4]. SJS and TEN are potentially fatal in acute phase due to the associated necrosis of external and internal body surfaces which predispose patients to life-threatening complications including sepsis and multi-organ failure. The mortality of SJS is less than 5%, whereas 30~50% TEN patients die of the acute phase of the disorder[2]. Severity of Illness Score for Toxic Epidermal Necrolysis (SCORTEN) score has been developed to evaluate the severity of TEN and predict its mortality[5]. Since this system is proven to be reliable and applicable for estimating risk for death among both SJS and TEN patients[6–8], doctors routinely calculate the score after the admission. Meanwhile, numerous studies set SCORTEN-derived expected mortality as internal control to evaluate the effects of immunomodulatory therapy on death prevention[9–12].

The pathogenesis of the disorder is still incompletely understood. Available evidences indicate that the synthesis of genetic susceptibility, antigen-specific immunity and mediators of cell death play key roles in the mechanism of the disease[13]. HLA-B*15:02 and HLA-A*31:01 have been implicated as risk factors after exposure to carbamazepine in the Han Chinese and Japanese, respectively[14, 15]. Despite the fact that genetic susceptibilities exist in specific racial groups, SJS/TEN is considered as a T cell mediated, type IV hypersensitivity disorder. Unlike most allergic skin reactions which CD4^+^ T cells are the predominant cell type, CD8^+^ T cells and NK cells concentrate in blister fluid and epidermis of SJS/TEN patients[13]. In terms of apoptosis mediators, some studies observe that Fas/Fas ligand (FasL) pathway participates in the keratinocyte death, a vital pathology of SJS/TEN[16–18]. This process is triggered by ligation of Fas on keratinocytes via membrane-bound or soluble FasL from T cells, mononuclear cells or keratinocytes themselves[2]. In addition, perforin/granzyme pathway is also involved. Once cytotoxic T cells recognize a target cell, perforin creates channels in the cell membrane, which allows granzyme B to enter the cell, activate the intracellular caspase cascade and result in apoptosis[19]. Additionally, granulysin, another cytotoxic molecule, is significantly increased in blister fluids of patients. Depleting granulysin reduces the cytotoxicity, while injection of this substance into mouse skin results in SJS/TEN mimicking features[20]. These biological evidences may provide us an insight that it is reasonable and promising to cure SJS/TEN by blocking these immunological processes.

Intravenous immunoglobulin (IVIG) is considered as a possible way to treat SJS/TEN due to its potential to suppress type IV hypersensitivity and hamper cell apoptosis. On one hand, IVIG leads to a decreased internalization inside antigen presenting cells and results in a reduced antigen-specific CD4^+^ T cell response[21]. On the other hand, CD8^+^ T cell activation and cytotoxic markers (perforin and CD107) are also strongly suppressed when therapeutic dose IVIG is given[22, 23]. In addition, there was evidence indicating that IVIG treatment could decrease the number of NK cells in peripheral blood and reduce the releasing of granzyme B into plasma[24]. Moreover, IVIG is capable to block Fas receptor and consequently protects keratinocytes from Fas-mediated cell death *in vitro*[16]. Actually, this medicine is commonly used in clinical treatment for SJS/TEN, however, no large randomized controlled trail is conducted to evaluate its efficiency due to the low incidence of SJS/TEN. Actually, some studies suggested IVIG could possibly reduce the time to heal and hospitalization[25–27]. According to a recent study, the mortality was decreased among IVIG treated SJS/TEN patients[28]. In line with that, an earlier systematic review reported a beneficial but insignificant clinical effect of IVIG[29]. While in another meta-analysis, the mortality of the high-dose group (total dose of IVIG ≥ 2 g/kg) was dramatically lower than that of the low-dose group among adults, however, this downward trend turned into insignificant in multivariate model[12]. Of note, this study showed the recovery time and the length of hospitalization were shorter in pediatric group compared with adult group[12].

Since other potentially protective therapies, such as corticosteroid[30], are often used concomitantly in clinical practice, the true impact of IVIG on SJS/TEN cases may be influenced. It is important to evaluate the differences between the solo and the combined regimen to facilitate clinicians to make optimized decisions. To examine whether IVIG alone or IVIG-corticosteroid combined therapy could accelerate the improvement of SJS/TEN, we reviewed studies that compared the time to recover in treated individuals against their counterparts. We also investigated whether IVIG therapy could reduce the mortality by combining studies that compared the actual mortality in treated cases against the expected death rate estimated from SCORTEN system.

## Materials and Methods

### Establishment of eligible criteria

There were no restrictions of age, sex and ethnicity. Two types of studies were included and analyzed separately as follow. On one hand, case-control studies that explored the effect of IVIG on the improvements of SJS/TEN patients were selected. Since most of these studies recruited individuals treated with the combination of IVIG and corticosteroid as case group and steroid applied cases as control group, only this kind of studies were selected. In addition, steroid therapy is widely used as a standard way to heal SJS/TEN[30], so it was reasonable to set it as a baseline and investigate the additive protective effect of IVIG. Two common variables, the time to arrest progression and the hospitalization length, were utilized to measure the improvements of patients. A small part of studies that reported the time of progression arrest by describing when fever control[9], skin healing[31] and re-epithelialization[32] occurred were also included, because these descriptions were supposed to reflect to what magnitude did IVIG treatment accelerate the improvement.

On the other hand, trails about SMR comparison between treated group and internal control group were accumulated. However, some of these were excluded for the studies contained less than five patients or did not observe a death patient, which made SMR calculation impossible. No restriction of treatment pattern was imposed. The remaining articles usually recorded total case numbers, SCORTEN scores upon admission and actual death numbers. Accordingly, expected mortality and SMR calculations could be given.

### Search of electronic databases

Literature search was conducted on PubMed, Web of Science, Cochrane Library, China Biology Medicine (CBM) disc, WanFang Database (Chinese) and Chinese National Knowledge Infrastructure (CNKI). The following key words or their equivalent Chinese terms were used to find all relative records: Stevens-Johnson syndrome, SJS, toxic epidermal necrolysis, TEN, immunoglobulin, intravenous immunoglobulin and IVIG. The period of literature search was from 1966 to Oct 2015. Reports were firstly screened according to titles and abstracts. The remaining trials were examined carefully by criterion and eligible ones were included.

### Extraction of data

Two reviewers read the included studies and extracted the following variables independently: first author, publication year, country, age (median, range), sex ratio (F/M), diagnosis (SJS, SJS/TEN overlap or TEN), detailed regimen, numbers of patients treated with different ways, time to arrest progression (days, mean ± SD), time to stay at hospital (days, mean ± SD), predicted mortality, actual mortality and SMR with 95% confidence interval (95% CI). Any disagreement was resolved by further consensus.

### Statistical analyses

The between-study heterogeneity was assessed by *Q*-statistic[33]. It was measured by *I*^*2*^ value, indicating the percent of the total variance across studies due to heterogeneity rather by chance. Heterogeneity was classified as high, medium and low when *I*^*2*^ ≥ 50%, 25% ≤ *I*^*2*^ < 50% or *I*^*2*^ < 25%, respectively[34]. The sources of inconsistencies within studies were investigated by meta-regression model. If no heterogeneity existed, quantitative data were pooled by Mantel–Haenszel’s method in fixed effect model, otherwise they were accumulated by Dersimonian and Laird method in random effect model[35]. Likewise, SMRs were also pooled in either random or fixed model dependent on whether the heterogeneity existed or not. Publication bias was assessed by the symmetry of funnel plot visually and by Egger’s linear regression test statistically[33]. Subgroup analyses were carried out to address whether the overall effect varied across different groups. Sensitivity analysis was performed to check the stability and reliability of pooled results by omitting each individual study. All statistical analyses were conducted by Stata 9.0 (Stata Crop LP, College station, TX). All *P* values were two-sided and identified as significant if less than 0.05.

## Results

### Eligible study selection

**Fig 1:**
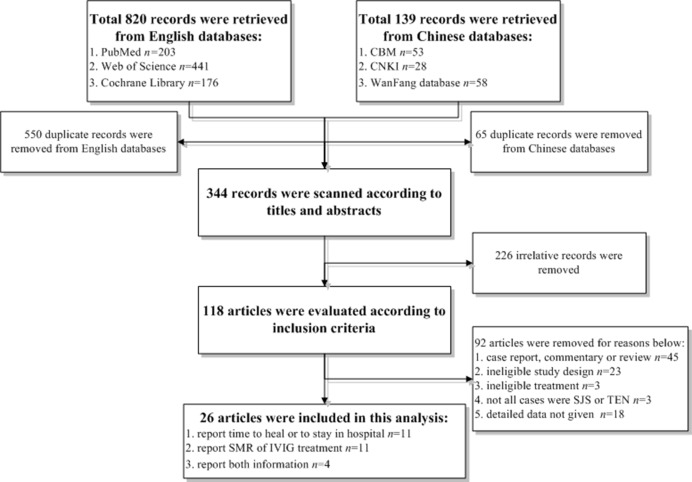
Flow diagram of eligible studies selection.

A total of 959 citations were retrieved according to the key words in the electronic databases. After dropping 615 duplicates, 344 records were left and scanned for titles and abstracts, which caused 226 irrelative citations removed. The remaining 118 articles were carefully examined according to eligible criteria and 92 of them were discarded for reasons showing in **Fig 1**, thus total 26 articles were ultimately included for analyses, in which 11 studies reported the recovery time, 11 studies reported SMR and 4 studies reported the both information.

### Study characteristics

Total 15 studies observed either cessation time or hospitalization length[9, 25–27, 31, 32, 36–44]. Of these, 3 studies recruited patients with SJS only[9, 36, 37], 5 studies chose TEN patients only[26, 31, 38–40] and 7 studies imposed no restrictions about patients recruitment[25, 27, 32, 41–44]. There were 628 individuals included in the analysis, which contained 317 controls applied with steroid only and 311 cases treated by combination of IVIG and corticosteroid. The exact regimen was summarized in **Table 1**. Ages varied with a range from below 1 to above 90. Most of these studies used median and range to describe them, however, a few articles[25, 40, 44] reported ages using mean and SD. With regards to sex ratio, it seemed to have a female predominance among SJS/TEN patients. Unfortunately, some studies[41–43] reported neither ages nor sex distributions clearly. (**Table 1**).

**Table 1:** Characteristics of studies about the effect of IVIG therapy on SJS/TEN recovery

Records	Age, years, median (range)	Sex ratio, F/M	Diagnose	IVIG therapy groups				Controls			
				*N*	Treatments	Time to arrest progression, days, mean ± SD	Hospital stay, days, mean ± SD	*N*	Treatments ^a^	Time to arrest progression, days, mean ± SD	Hospital stay, days, mean ± SD
Ahluwalia[9] 2014 USA	10 (7–14)^#^	5/5	SJS	6	Total 2–2.5 g/kg IVIG alone or with total 4 mg/kg corticosteroids	1.8 ± 0.9*	12.5 ± 2.9*	4	total 4 mg/kg corticosteroids or supportive therapy	0.8± 1.0*	6.3± 2.4*
Chen[25] 2010 China	37 ± 16	40/42	SJS/TEN	24	Total 0.7–7.4 g/kg IVIG over 3–15 days with corticosteroid therapy	NG	18.1 ± 5.3	58	corticosteroid therapy	NG	26.4 ± 9.5
Dai[41] 2012 China	NG	21/19	SJS/TEN	20	Total 1.2–2.0 g/kg IVIG with MP	4.7 ± 1.3	14.0 ± 5.9	20	MP treatment	4.9 ± 1.0	16.2 ± 5.5
Jagadeesan[26] 2013 India	37 (6–68)	20/16	TEN	18	Total 0.2–0.5 g/kg IVIG with 0.1–0.3 mg/(kg*d) dexamethasone	3.9 ± 1.9	13.3 ± 5.4	18	0.1–0.3 mg/(kg*d) dexamethasone	5.9 ± 1.4	15.3 ± 6.2
Jin[42] 2011 China	NG	49/33	SJS/TEN	41	Total 0.6–1.0 g/kg IVIG with 0.5–1.0 mg/(kg*d) betamethasone	2.4 ± 0.5	27.3 ± 1.6	41	0.5–1.0 mg/(kg*d) betamethasone	4.8 ± 0.5	34.7 ± 1.6
Lalosevic[32] 2014 Serbia	42 (1–94)	21/17	SJS/TEN	6	2.0 g/(kg*d) IVIG with 1–2 mg/(kg*d) MP	8.8 ± 2.3	31.6 ± 5.4	8	1–2 mg/(kg*d) MP	9.0 ± 2.9	27.9 ± 7.8
Liu[43] 2012 China	NG	44/38	SJS/TEN	35	Total 0.6–2 g/kg IVIG with 1–2 mg/(kg*d) prednisone	NG	18.6 ± 5.2	34	1–2 mg/(kg*d) prednisone	NG	21.4 ± 5.4
Lu[44] 2013 China	SJS:38.4± 19.2; TEN:41.3 ± 22.2	35/27	SJS/TEN	30	Total 1.2–2.0 g/kg IVIG with 1.5–3.0 mg/(kg*d) MP	4.4 ± 0.92	NG	32	1.5–3.0 mg/(kg*d) MP	5.8 ± 2.0	NG
Stella[31] 2007 Italy	66 (26–86)	23/8	TEN	23	Total 2.8 g/kg IVIG with 250 mg MP every 6 h for the first 48h	5.1 ± 2.0	16.4 ± 5.5	8	corticosteroid	4.2 ± 1.9	11.8 ± 6.0
Wang[38] 2010 China	32.4^§^ (6–82)	6/9	TEN	14	Total 2.0 g/kg IVIG for 5 days with 2 mg/(kg*d) prednisone	3.4 ± 1.2	24.5 ± 8.4	11	2 mg/(kg*d) prednisone	8.7 ± 2.5	33.9 ± 7.8
Xia[39] 2012 China	43.51^§^ (11–65)	20/15	TEN	9	Total 2.0–4.0 g/kg IVIG and 60–80 mg/d MP	NG	22.4 ± 7.4	7	60–80 mg/d MP	NG	17.1 ± 5.7
Yang[27] 2009 China	40.5 (16–79)	10/8	SJS	7	Total 2.0 g/kg IVIG with 1–1.5 mg/(kg*d) MP	4.3 ± 2.6	19.7 ± 4.4	8	1–1.5 mg/(kg*d) MP	7.0 ± 2.8	29.0 ± 10.6
	42.5^§^ (6–86)	22/25	TEN	10	Total 2.0 g/kg IVIG with 1–1.5 mg/(kg*d) MP	4.3 ± 2.4	23.4 ± 5.1	27	1–1.5 mg/(kg*d) MP	7.2± 3.4	34.3 ± 16.0
Zhang[36] 2013 China	5 (1–12)	11/19	SJS	17	Total 2.0–4.0 g/kg IVIG with 10–30 mg/d MP	1.6 ± 0.7	11.9 ± 7.4	13	10–30 mg/d MP	2.7± 0.8	23.3 ± 14.8
Jiang[37] 2007 China	(1.5–13)	9/15	SJS	12	Total 2.0 g/kg IVIG with 0.5–1.0 mg/(kg*d) dexamethasone	3.0 ± 1.2	NG	12	0.5–1.0 mg/(kg*d) dexamethasone	6.0 ± 1.4	NG
Zhu[40] 2012 China	46 ± 19	26/35	TEN	39	Total 2.0 g/kgIVIG with 1.5 mg/(kg*d) MP for 3–5 days	7.6 ± 2.7	22.6 ± 13.7	16	1.5 mg/(kg*d) MP for 3–5 days	12.3 ± 10.1	28.6 ± 29.9

SJS, Stevens-Johnson syndrome; TEN, toxic epidermal necrolysis; IVIG, intravenous immunoglobulin; MP, methylprednisolone; NG, not given.

^a^ the period of treatment varied as individual situation unless otherwise mentioned.

^#^ interquartile range.

^§^ mean instead of median.

*mean and SD were transformed from sample size, median and range according to Hozo et al, 2005.

There were 17 studies investigated the effect of IVIG on SMR reduction[10, 11, 25–27, 31, 40, 43, 45–53]. Of these, 9 selected TEN patients[26, 31, 40, 45–50] and 8 recruited patients without restriction[10, 11, 25, 27, 43, 51–53], which accumulated 361 cases treated by IVIG within this analysis. In terms of therapy, 167 patients were given solo IVIG treatment[10, 11, 46, 47, 49–52], 189 patients were treated with combination of IVIG and steroid[25–27, 31, 40, 43, 45, 53], and 5 patients were treated with IVIG and plasmapheresis[48]. Some articles also reported the time to recover or in hospital with no control to compare with[50, 52, 53]. (**Table 2**)

**Table 2:** Characteristics of studies about the effect of IVIG therapy on SJS/TEN mortality

Records	Age, median (range)	Sex ratio, F/M	Diagnosis	*N* ^a^	Treatments	Predicted mortality	Observed mortality	SMR (95% CI)	Time to arrest progression, days, mean ± SD	Hospital stay, days, mean ± SD
Bachot[51] 2003 France	40 (13–88)	22/19	SJS/TEN	34	Total 2 g/kg IVIG	8.252	11	1.33 (0.65–2.39)	NG	NG
Brown[45] 2004 USA	45 ± 25	23/22	TEN	24	Total 1.6 g/kg IVIG with corticosteroid in certain cases	7.713	10	1.30 (0.61–2.39)	NG	NG
Campione[46] 2003 Italy	42(12–95)	8/2	TEN	10	Total 2 g/kg IVIG	3.201	1	0.31 (0.03–1.75)	NG	NG
Chen[25] 2010 China	37 ± 16	40/42	SJS/TEN	24	Total 2.7 ± 1.5 g/kg IVIG after corticosteroid treatment	5.277	3	0.57(0.11–1.67)	NG	18.1 ± 5.3
Jagadeesan[26] 2013 India	37 (6–68)	20/16	TEN	18	Total 0.2–0.5 g/kg IVIG with 0.1–0.3 mg/(kg*d) dexamethasone	5.49	1	0.18(0.02–1.02)	3.9 ± 1.9	13.3 ± 5.4
Kim[47] 2005 Korea	44.8^§^ (2–80)	18/20	TEN	14	Total 1.6–2.0 g/(kg) IVIG	2.353	1	0.42 (0.04–2.38)	NG	NG
Lee[10] 2013 Singapore	57 ± 19	38/26	SJS/TEN overlap and TEN	64	Total 2.4±0.8 g/(kg) IVIG	18.22	20	1.10 (0.67–1.69)	NG	NG
Lissia[48] 2005 Italy	74 (39–87)	1/4	TEN	5	Total 3.0 g/kg IVIG for the first 3 days, and total 1.5 g/kg IVIG for the next 3 days, combined with plasmapheresis	3.319	1	0.30 (0.03–1.69)	2.8 ± 0.8	17.6 ± 12.0
Liu[43] 2012 China	NG	44/38	SJS/TEN	35	Total 0.6–2.0 g/kg IVIG with 1–2 mg/(kg*d) prednisone	9.231	5	0.54(0.17–1.27)	NG	18.6 ± 5.2
Rajaratanam[49] 2010 UK	54 (18–86)	13/8	TEN	14	Total 0.8–5.0 g/kg IVIG	5.021	3	0.60 (0.12–1.75)	4.5 ± 1.5	NG
Stella[31] 2007 Italy	66 (26–86)	23/8	TEN	23	Total 2.8 g/kg IVIG with MP at doses of 0.25 g every 6 h for the first 48 h of admission	8.244	6	0.73 (0.27–1.59)	5.0 ± 2.0	14.3 ± 6.0
Tan[11] 2012 Singapore	48.5 (13–85)	14/14	SJS/TEN overlap and TEN	9	Total 3.0 g/kg IVIG	2.386	1	0.42 (0.04–2.35)	NG	16.0 ± 4.2
Teo[52] 2009 Singapore	51.5 (30–84)	5/1	SJS/TEN	6	Total 3.0 g/kg IVIG	1.155	1	0.87 (0.09–4.85)	3.2 ± 1.3	36.5 ± 21.7
Trent[50] 2003 USA	45 (19–62)	8/8	TEN	16	Total 4.0 g/kg IVIG	5.808	1	0.17 (0.02–0.96)	3.8 ± 3.8	20.3 ± 13.7
Yang[27] 2009 China	40 (15–86)	32/33	SJS/TEN	20	Total 2.0 g/kg IVIG with corticosteroid	3.51	3	0.85 (0.17–2.51)	NG	NG
Yeung[53] 2005 China	57.5 (18–87)	3/3	SJS/TEN	6	Total 3.0 g/kg IVIG with corticosteroid in certain cases	2.257	1	0.44 (0.04–2.48)	4.9 ± 1.7	17.3 ± 9.5
Zhu[40] 2012 China	46 ± 19	26/35	TEN	39	Total 2.0 g/kg IVIG with 1.5 mg/(kg*d) MP for 3–5 days	9.341	5	0.54(0.17–1.25)	7.6 ± 2.7	22.6 ± 13.7

SJS, Stevens-Johnson syndrome; TEN, toxic epidermal necrolysis; SMR, standardized mortality ratio; IVIG, intravenous immunoglobulin; MP, metilprednisolone; NG, not given.

^§^mean instead of median.

^a^ the number of individuals that were actually included in the analysis.

### IVIG treatment significantly accelerated the improvement of SJS/TEN patients

**Fig 2:**
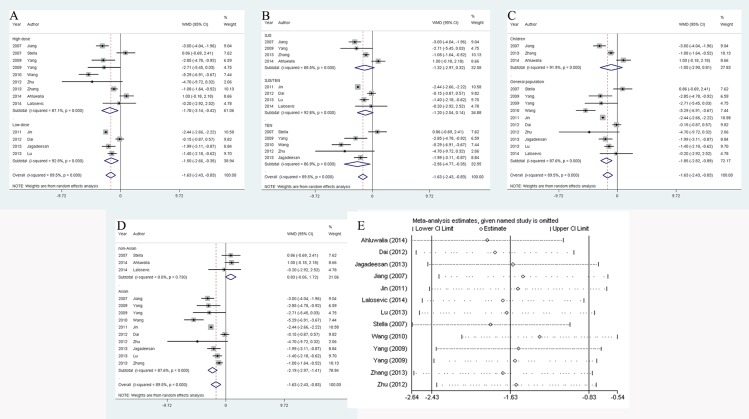
Analysis and assessment of the effect of combination therapy on SJS/TEN recovery. A: forest plot of the impact of combined therapy stratified by IVIG dose; B: forest plot of the impact of combined therapy stratified by diagnosis; C: forest plot of the impact of combined therapy stratified by age; D: forest plot of the impact of combined therapy stratified by area; E: sensitivity analysis to check the reliability of the pooled result.

In general, IVIG combined with corticosteroid therapy markedly reduced the cessation time against steroid therapy by **1.63 (95% CI: 0.83–2.43, *P* < 0.001)** days (**Fig 2A**). This result was proven to be stable by sensitivity analysis (**Fig 2E**), however major difference between studies was detected (*I*^*2*^ = 89.5%, *P* < 0.001). Therefore we investigated the possible sources of heterogeneity by conducting subgroup analyses. IVIG dose (high dose and low dose), diagnosis (SJS, SJS/TEN overlap and TEN), age (children and general population) and area (Asian and non-Asian) were the potential sources of it. In detail, total IVIG dose ≥ 2.0 g/kg was referred as high dose, others were defined as low dose. Of note, some Chinese studies reported the upper limit of IVIG usage was 2.0 g/kg[41, 42, 44], which indicated a routine low dose administration and were classified as low dose subgroup. Additionally, Asian group was categorized as Chinese, Singaporean, Korean and Indian populations. The remaining studies on American, French, Italian, British and Serbian populations were classified as non-Asian group.

Subgroup analyses indicated the benefit of IVIG combined with corticosteroid therapy was restricted to general population (against children), TEN patients and Asian population (**Fig 2B, 2C and 2D**). Besides, this favorable effect was significant regardless of IVIG dosage (**Fig 2A**). However, difference within studies still existed in all subgroups except for analysis stratified by area, in which heterogeneity was disappeared in non-Asian subgroup (*I*^*2*^ = 0.0%, *P* = 0.73). But in non-Asian subgroup, it was demonstrated that the combination treatment had the trend to prolong the time. In order to confirm that area was the source of inconsistency, we conducted a meta regression analysis and chose area, publication year, diagnosis, IVIG dose and age as covariates. As a result, only regional factor had an great influence on consistency within studies (*t* = 3.53, *P* = 0.01) (**Table 3**).

**Table 3:** Meta regression analysis: the effect of potential covariables on arrest of progression

Variables*	Regression coefficient	Standard error	95% CI	*t*	*P*
**Area**	**3.72**	**1.05**	**1.23–6.21**	**3.53**	**0.010**
Publication year	0.02	0.18	-0.42–0.45	0.11	0.918
Age	-0.65	1.57	-4.35–3.05	-0.42	0.689
IVIG dose	-1.92	1.14	-4.62–0.79	-1.68	0.138
Diagnosis	-0.48	0.77	-2.30–1.34	-0.62	0.552

*String variables were converted into numeric values by the following rules: Area: 1 Asian, 2 on-Asian; age: 1 children, 2 general population; IVIG dose: 1 low dose, 2 high dose; Diagnosis: 1 SJS, 2 mix of SJS and TEN, 3 TEN.

**Fig 3:**
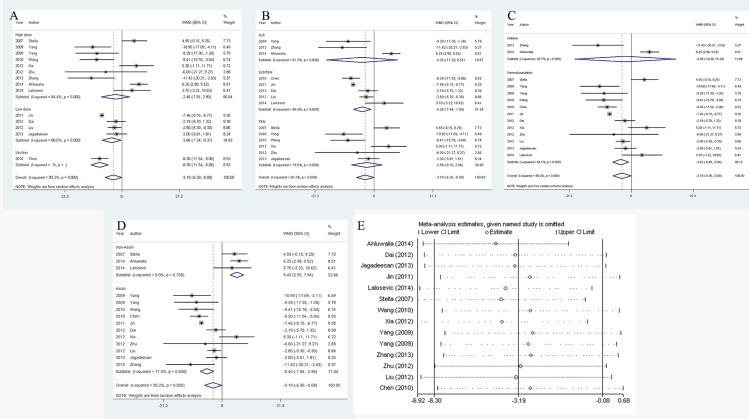
Analysis and assessment of the effect of combination therapy on SJS/TEN admission length. A: forest plot of the impact of combined therapy stratified by IVIG dose; B: forest plot of the impact of combined therapy stratified by diagnosis; C: forest plot of the impact of combined therapy stratified by age; D: forest plot of the impact of combined therapy stratified by area; E: sensitivity analysis to check the reliability of the pooled result.

With respect to the length of admission for SJS/TEN patients, the overall effect of combination therapy could shorten by **3.19** days **(95% CI: 0.08–6.30, *P* = 0.045)** against steroid only (**Fig 3A**). Unfortunately, this result was not reliable according to sensitivity analysis (**Fig 3E**). Removal of 9 studies[25–27, 36, 38, 40–43] would reverse the significant overall effect to insignificant one. Of note, these 9 studies were all from Asia. Furthermore, the pooled outcome of non-Asian group showed that combined regimen prolonged hospitalization length (5.43 (95% CI: 2.93–7.94, *P* < 0.001)) (**Fig 3D**). In terms of heterogeneity detection, remarkable inconsistencies within studies were found in overall analysis (*I*^*2*^ = 90.2%, *P* < 0.001) and in all subgroups except non-Asian population (*I*^*2*^ = 0.0%, *P* = 0.74). Similarly, we employed meta regression model and certified area was the source of the inconsistency (*t* = 3.84, *P* = 0.005). (**Table 4**).

**Table 4:** Meta regression analysis: the effect of potential covariables on hospitalization length.

Variables*	Regression coefficient	Standard error	95% CI	*t*	*P*
**Area**	**11.79**	**3.07**	**4.71–18.87**	**3.84**	**0.005**
Publication year	1.13	0.73	-0.57–2.82	1.53	0.165
Age	1.78	5.28	-10.38–13.95	0.34	0.744
IVIG dose	-1.03	1.98	-5.61–3.54	-0.52	0.617
Diagnosis	2.01	2.09	-2.81–6.84	0.96	0.364

*String variables were converted into numeric values by the following rules: Area: 1 Asian, 2 non-Asian; age: 1 children, 2 general population; IVIG dose: 1 low dose, 2 high dose; Diagnosis: 1 SJS, 2 mix of SJS and TEN, 3 TEN.

### IVIG treatment had a trend to reduce mortality among SJS/TEN patients

**Fig 4:**
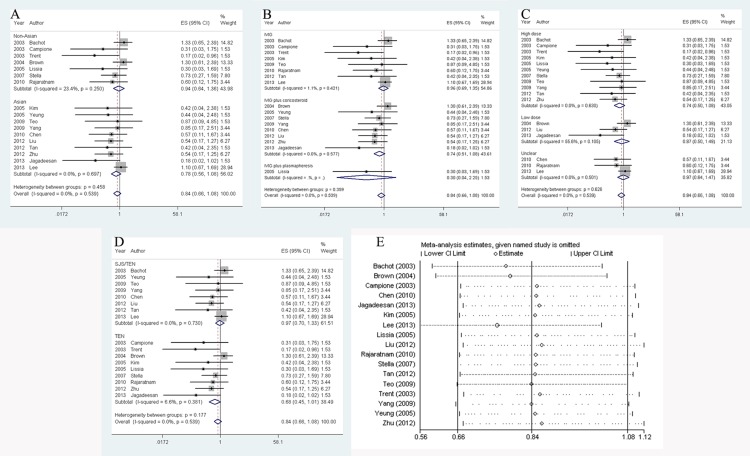
Analysis and assessment of the effect of IVIG therapy on SJS/TEN SMR. A: forest plot of the impact of IVIG therapy stratified by different areas; B: forest plot of the impact of IVIG therapy stratified by different therapeutic patterns; C: forest plot of the impact of IVIG therapy stratified by IVIG dose; D: forest plot of the impact of combined therapy stratified by diagnosis; E: sensitivity analysis to check the reliability of the pooled results.

As shown in **Fig 4**, IVIG therapy reduced the mortality by 16%. However, this outcome was not statistically significant (95% CI: 0.66–1.08, *P* = 0.178). No heterogeneity was found (*I*^*2*^ = 0.0%, *P* = 0.539). In order to investigate if the benefit of IVIG administration was restricted to certain subgroups, we stratified included studies by area, regimen, IVIG dose and diagnosis. Generally, no sub-analyses demonstrated results that were statistically significant. Of note, it seemed that IVIG treatment would have a greater effect on patients with TEN since it reduced the mortality by 32% (95% CI: 0.45–1.01, *P* = 0.06) (**Fig 4D**). Despite that, other subgroup analyses also showed possibly favorable outcomes that were slightly inferior to the effect on TEN patients. As results, high IVIG dose administration lowered the mortality by 26% (95% CI: 0.50–1.08, *P* = 0.116), and low dose only lowered it by 13% (95% CI: 0.50–1.49, *P* = 0.608) (**Fig 4C**). In addition, combination of IVIG and steroid reduced mortality by 26% (95% CI: 0.51–1.08, *P* = 0.118), while solo IVIG usage decreased it by 4% (95% CI: 0.69–1.35, *P* = 0.819) (**Fig 4B**). Besides, IVIG decreased mortality by 22% and 6% in Asian (95% CI: 0.56–1.08, *P* = 0.134) and non-Asian cases (95% CI: 0.64–1.36, *P* = 0.736), respectively (**Fig 4A**).

### Detection of publication bias

**Fig 5:**
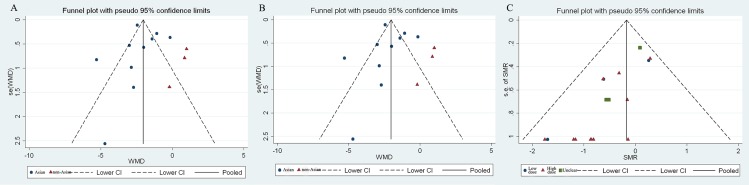
Detection of publication bias by evaluating the symmetry of funnel plot. A: funnel plot test on studies regarding the impact of combined therapy on SJS/TEN recovery; B: funnel plot test on studies regarding the impact of combined therapy on the time to stay in hospital; C: funnel plot test on studies regarding the impact of IVIG therapy on SJS/TEN mortality.

The graphic asymmetry of funnel plot suggested obvious publication bias existed in three analyses above (**Fig 5**). The asymmetries were measured by Egger’s linear regression and a significant bias was detected in analysis about the effect of IVIG on accelerating recovery (*z* = -1.42, *P* < 0.001), however, no bias was detected in regional subgroup studies (Asian: *z* = 0.24, *P* = 0.856; non-Asian: *z* = -1.49, *P* = 0.121)(**Fig 5A**). Similarly, as illustrated in **Fig 5B**, publication bias was found in overall study (*z* = -0.89, *P* = 0.031), but not in stratified investigations (Asian: *z* = 0.93, *P* = 0.285; non-Asian: *z* = -1.51, *P* = 0.195). Remarkable bias was also detected in terms of the study on SMR (*z* = -1.42, *P* < 0.001) (**Fig 5C**), while in low dose subgroup, publication bias disappeared (*z* = -1.49, *P* = 0.121).

## Discussion

SJS and TEN are the same type of adverse drug hypersensitivity with different extents of skin lesion[1, 2]. It is well established that drug specific T cell is a principal contributor to the pathogenesis of this disease. The biologic process is majorly mediated by activated cytotoxic T lymphocytes (CD8^+^ T cells in most cases) via generating FasL or perforin/granzyme and resulting in keratinocytes death. In addition, activated CD4^+^ T cells also participate in this by disrupting cytokine production[2]. Despite cutaneous lesion, sepsis and other organ involvements also occur among many SJS/TEN patients, which causes worse prognoses[54]. Although the mechanism of these co-morbidities are not fully elucidated, there is a strong possibility that they are driven by elicitation of inflammatory pathways[55]. These evidences give us an insight into treating SJS/TEN by correcting immune dysfunction.

The possible protective effect of IVIG on SJS/TEN patients is substantially attributed to blockade of FasL. It is well known that SJS/TEN is characterized by massive apoptosis of keratinocyte and increased expression of FasL in serum and epidermis[16]. Viard et al. [16] found IVIG, which dosage was equivalent to clinical use, could completely inhibit keratinocyte death caused by human recombinant FasL *in vitro*. This protection was mediated by naturally occurring anti-Fas immunoglobulin via neutralizing Fas receptor[16]. In addition, some findings are helpful complements on pathological mechanism, revealing that IVIG is capable to suppress type IV hypersensitivity and vital cytotoxic markers production. Meanwhile, it has been demonstrated that IVIG treatment decreases the number of NK cells in peripheral blood and the releasing of granzyme B into plasma[24].

We explore whether IVIG, a common immune modulator, could heal SJS/TEN. Overall, there is a cluster of reliable evidences show beneficial roles of IVIG on the improvement of SJS/TEN patients. Firstly, the combination of IVIG and corticosteroid reduced cessation time significantly (by 1.63 days). Moreover, this favorable effect became stronger among Asian or TEN patients (reduced time by 2.19 or 2.56 days, respectively). Meanwhile, high dose IVIG administration (total dose ≥ 2 g/kg) could slightly strengthen this protection (reduced time by 1.78 days). Heterogeneity was detected in overall analysis and we tried to identify the source of heterogeneity by stratified analysis. In non-Asian subgroup, no heterogeneity within studies was found, whereas heterogeneity still persisted within Asian subgroup, suggesting that regional factor was likely to be one of the sources. This proposal was further validated by meta-regression analysis. Secondly, the combination of IVIG and corticosteroid decreased the hospitalization length by 3.19 days in general, although this result was not reliable due to the fact of an unstable outcome in sensitivity analysis. Besides, subgroup analysis indicated that findings of Asian and non-Asian groups were completely opposite, showing a favorable effect among Asian and a detrimental effect among non-Asian.

The difference within studies is possibly caused by reporting bias, since among studies from China, there was higher proportion of positive results reported. 75% (6/8) of the included Chinese studies described the combination regimen accelerated the recovery time. Despite that, geographical diversity may bring other issues, for example, the severity of hospitalized cases may vary across areas. It is common that doctors from Asian countries, for instance China, treat each SJS/TEN case with combined therapy. Whereas, this regimen seems to be restricted to individuals who suffer from more severe illness in non-Asian countries, because 85.7% (6/7) of the non-Asian studies recruited only TEN patients. According to SCORTEN system, the expected mortality of SJS patients with score 1 is 3.2%, and the rate of TEN patients with score of 5 sharply rises to 90%[5]. Therefore, severe patients, who are more likely to be recruited in non-Asian studies, are expected to have worse prognoses, although combined treatment is applied. This proposal may explain why three studies conducted from non-Asian countries [9, 31, 32] consistently reported longer cessation time and admission length.

Given the rarity of SJS/TEN, the majority of the published articles investigating the effect of IVIG on SJS/TEN associated sequelae are conflicting retrospective case reports or series. Therefore, evidence-based recommendations are difficult to generate. In terms of ocular complications of SJS/TEN, it is indicated that the application of IVIG during the acute phase did not appear to diminish the severity of ocular complications (n = 43)[56]. Similarly, a series of 8 TEN patients treated with IVIG at 2 g/kg over 2 days did not show better effect than a historical control group (n = 18)[57]. In contrast, A beneficial effect was observed among those who were given IVIG within 6 days of disease onset[58]. In terms of SJS/TEN associated respiratory disorders, relevant studies are limited. Furthermore, no special attention is paid to the effect of IVIG or other systemic therapies in most of these studies. On the contrary, the distribution of IVIG application among SJS/TEN patients requiring mechanical ventilation or not was described[59]. Likewise, another study reported the distribution of cyclosporine treatment among delayed pulmonary dysfunction patients with and without diffusion impairment[60]. A Japanese patient with SJS developed progressive dyspnea attacks after administration of betamethasone over one month. Although treated with corticosteroid, the patient died one year and seven months later because of the ultimate obliterative bronchitis[61]. Overall, published data provide few but controversial evidence for the effect of IVIG or other systemic therapies on SJS/TEN associated sequelae. Thus, no clear guidelines are generated and more investigations are warranted.

As mentioned above, sepsis and liver injury are the most common life-threatening complications of SJS/TEN[49]. Recent infection or concomitant hepatic dysfunction could increase the risk of death by as high as 2 folds for SJS/TEN patients[62]. Clinically, IVIG is capable to attenuate sepsis and immunological liver injury[63, 64]. In vitro, protective effect of IVIG is observed in liver and immune cells[16]. So intervention of serious complication by IVIG could be a theoretically plausible way to reduce the mortality. We investigated whether IVIG could improve survival by data synthesis. As predicted, pooled outcome suggested a favorable but statistically insignificant effect (SMR: 0.84, 95% CI: 0.66–1.08, *P* = 0.178). Since corticosteroid application might be a confounding factor, we stratified the studies based on the prescription. Results showed that combination therapy trended to reduce mortality by 26%, which was more profound than the potential effect of solo IVIG therapy (reduced mortality by 4%). This indicated steroid was crucial and should be taken into account when evaluating the effect of IVIG therapy. In addition, we found high dose IVIG administration (total dose ≥ 2g/kg) was likely to be more beneficial (SMR: 0.74, 95% CI: 0.50–1.08, *P* = 0.116). These statistically insignificant outcomes are supported by the National Institute for Health and Care Excellence (NICE) accredited review of the acute management of TEN published in 2016[65] which carefully reviewed the available data and considered that there was no conclusive evidence to make specific recommendations for or against IVIG application. Therefore, the group considered that IVIG should be practiced under the supervision of a specialist in a skin failure multidisciplinary team in the context of a clinical study or a case registry.

The results in this meta-analysis should be interpreted with caution for some limitations. First, heterogeneities and publication biases were detected. This is usually caused by reporting bias since positive outcomes are more likely to be published, especially for Asian studies. In the analyses investigating IVIG on SJS/TEN improvement, we found that 72.7% (8/11) of the Chinese studies reported positive outcomes, whereas 0 (0/4) of the non-Chinese studies reported positive outcomes. In terms of heterogeneity, we identified the source of it as area by subgroup analyses and meta regression and it disappeared in non-Asian group. Similarly, we controlled the publication bias by regional stratification. Second, we failed to evaluate the effects of solo IVIG treatment on recovery and hospitalization due to limitations of wanted reports. For example, few trails chose supportive therapy as control to estimate the effect of solo IVIG therapy. As we all known, solo IVIG treatment is routinely used in many western countries, so more high quality random controlled trails are further needed. Likewise, we were unable to identify if there was an additive or synergistic interaction between IVIG and corticosteroid by assessing to what extent corticosteroid application made a contribution to the impact of combination therapy on SMR. Third, we excluded studies on SMR with no mortality in the treatment group and the pooled result might underestimate the treatment effect consequently. So it is still promising to reduce death by using high dose IVIG among Asian population, since the results from these two sub-analyses were marginally insignificant.

In conclusion, IVIG combined with corticosteroid treatment shortens the recovery time of SJS/TEN patients. The effect is greater among Asian individuals. Furthermore, IVIG therapy is likely to reduce mortality of this disease. However, this outcome needs more high-quality studies to be confirmed.

## Supporting Information

S1 File Full eletronic search strategy for PubMed. (DOC)

S2 File PRISM 2009 checklist. (DOC)

## References

[1] Bastuji-Garin S, Rzany B, Stern RS, Shear NH, Naldi L, Roujeau JC. Clinical classification of cases of toxic epidermal necrolysis, Stevens-Johnson syndrome, and erythema multiforme. Arch Dermatol. 1993;129(1): 92–6. 8420497

[2] Borchers AT, Lee JL, Naguwa SM, Cheema GS, Gershwin ME. Stevens-Johnson syndrome and toxic epidermal necrolysis. Autoimmun Rev. 2008;7(8): 598–605. 10.1016/j.autrev.2008.06.004 18603022

[3] Chang VS, Chodosh J, Papaliodis GN. Chronic Ocular Complications of Stevens-Johnson Syndrome and Toxic Epidermal Necrolysis: The Role of Systemic Immunomodulatory Therapy. Semin Ophthalmol. 2016;31(1–2): 178–87. 10.3109/08820538.2015.1114841 26959145

[4] Saeed H, Mantagos IS, Chodosh J. Complications of Stevens-Johnson syndrome beyond the eye and skin. Burns. 2016;42(1): 20–7. 10.1016/j.burns.2015.03.012 25865527

[5] Bastuji-Garin S, Fouchard N, Bertocchi M, Roujeau JC, Revuz J, Wolkenstein P. SCORTEN: a severity-of-illness score for toxic epidermal necrolysis. J Invest Dermatol. 2000;115(2): 149–53. 10.1046/j.1523-1747.2000.00061.x 10951229

[6] Cartotto R, Mayich M, Nickerson D, Gomez M. SCORTEN accurately predicts mortality among toxic epidermal necrolysis patients treated in a burn center. J Burn Care Res. 2008;29(1): 141–6. 10.1097/BCR.0b013e31815f3865 18182912

[7] Guegan S, Bastuji-Garin S, Poszepczynska-Guigne E, Roujeau JC, Revuz J. Performance of the SCORTEN during the first five days of hospitalization to predict the prognosis of epidermal necrolysis. J Invest Dermatol. 2006;126(2): 272–6. 10.1038/sj.jid.5700068 16374461

[8] Bansal S, Garg VK, Sardana K, Sarkar R. A clinicotherapeutic analysis of Stevens-Johnson syndrome and toxic epidermal necrolysis with an emphasis on the predictive value and accuracy of SCORe of Toxic Epidermal Necrolysis. Int J Dermatol. 2015;54(1): e18–26. 10.1111/ijd.12466 25534407

[9] Ahluwalia J, Wan J, Lee DH, Treat J, Yan AC. Mycoplasma-associated Stevens-Johnson syndrome in children: retrospective review of patients managed with or without intravenous immunoglobulin, systemic corticosteroids, or a combination of therapies. Pediatr Dermatol. 2014;31(6): 664–9. 10.1111/pde.12481 25424206

[10] Lee HY, Lim YL, Thirumoorthy T, Pang SM. The role of intravenous immunoglobulin in toxic epidermal necrolysis: a retrospective analysis of 64 patients managed in a specialized centre. Br J Dermatol. 2013;169(6): 1304–9. 10.1111/bjd.12607 24007192

[11] Tan SK, Tay YK. Profile and pattern of Stevens-Johnson syndrome and toxic epidermal necrolysis in a general hospital in Singapore: treatment outcomes. Acta Derm Venereol. 2012;92(1): 62–6. 10.2340/00015555-1169 21710108

[12] Huang YC, Li YC, Chen TJ. The efficacy of intravenous immunoglobulin for the treatment of toxic epidermal necrolysis: a systematic review and meta-analysis. Br J Dermatol. 2012;167(2): 424–32. 10.1111/j.1365-2133.2012.10965.x 22458671

[13] Kohanim S, Palioura S, Saeed HN, Akpek EK, Amescua G, Basu S, et al Stevens-Johnson Syndrome/Toxic Epidermal Necrolysis—A Comprehensive Review and Guide to Therapy. I. Systemic Disease. Ocul Surf. 2016;14(1): 2–19. 10.1016/j.jtos.2015.10.002 26549248

[14] Chung WH, Hung SI, Hong HS, Hsih MS, Yang LC, Ho HC, et al Medical genetics: a marker for Stevens-Johnson syndrome. Nature. 2004;428(6982): 486 10.1038/428486a 15057820

[15] Ozeki T, Mushiroda T, Yowang A, Takahashi A, Kubo M, Shirakata Y, et al Genome-wide association study identifies HLA-A*3101 allele as a genetic risk factor for carbamazepine-induced cutaneous adverse drug reactions in Japanese population. Hum Mol Genet. 2011;20(5): 1034–41. 10.1093/hmg/ddq537 21149285

[16] Viard I, Wehrli P, Bullani R, Schneider P, Holler N, Salomon D, et al Inhibition of toxic epidermal necrolysis by blockade of CD95 with human intravenous immunoglobulin. Science. 1998;282(5388): 490–3. 9774279 10.1126/science.282.5388.490

[17] Viard-Leveugle I, Gaide O, Jankovic D, Feldmeyer L, Kerl K, Pickard C, et al TNF-alpha and IFN-gamma are potential inducers of Fas-mediated keratinocyte apoptosis through activation of inducible nitric oxide synthase in toxic epidermal necrolysis. J Invest Dermatol. 2013;133(2): 489–98. 10.1038/jid.2012.330 22992806

[18] Fu M, Gao Y, Pan Y, Li W, Liao W, Wang G, et al Recovered patients with Stevens-Johson syndrome and toxic epidermal necrolysis maintain long-lived IFN-gamma and sFasL memory response. PLoS One. 2012;7(9): e45516 10.1371/journal.pone.0045516 23029066 PMC3445504

[19] Bolitho P, Voskoboinik I, Trapani JA, Smyth MJ. Apoptosis induced by the lymphocyte effector molecule perforin. Curr Opin Immunol. 2007;19(3): 339–47. 10.1016/j.coi.2007.04.007 17442557

[20] Chung WH, Hung SI, Yang JY, Su SC, Huang SP, Wei CY, et al Granulysin is a key mediator for disseminated keratinocyte death in Stevens-Johnson syndrome and toxic epidermal necrolysis. Nat Med. 2008;14(12): 1343–50. 10.1038/nm.1884 19029983

[21] Aubin E, Lemieux R, Bazin R. Indirect inhibition of in vivo and in vitro T-cell responses by intravenous immunoglobulins due to impaired antigen presentation. Blood. 2010;115(9): 1727–34. 10.1182/blood-2009-06-225417 19965673

[22] Trepanier P, Bazin R. Intravenous immunoglobulin (IVIg) inhibits CD8 cytotoxic T-cell activation. Blood. 2012;120(13): 2769–70. 10.1182/blood-2012-07-445007 23019205

[23] Trepanier P, Chabot D, Bazin R. Intravenous immunoglobulin modulates the expansion and cytotoxicity of CD8+ T cells. Immunology. 2014;141(2): 233–41. 10.1111/imm.12189 24128001 PMC3904244

[24] Jacobi C, Claus M, Wildemann B, Wingert S, Korporal M, Romisch J, et al Exposure of NK cells to intravenous immunoglobulin induces IFN gamma release and degranulation but inhibits their cytotoxic activity. Clin Immunol. 2009;133(3): 393–401. 10.1016/j.clim.2009.09.006 19828380

[25] Chen J, Wang B, Zeng Y, Xu H. High-dose intravenous immunoglobulins in the treatment of Stevens-Johnson syndrome and toxic epidermal necrolysis in Chinese patients: a retrospective study of 82 cases. Eur J Dermatol. 2010;20(6): 743–7. 10.1684/ejd.2010.1077 20952352

[26] Jagadeesan S, Sobhanakumari K, Sadanandan SM, Ravindran S, Divakaran MV, Skaria L, et al Low dose intravenous immunoglobulins and steroids in toxic epidermal necrolysis: a prospective comparative open-labelled study of 36 cases. Indian J Dermatol Venereol Leprol. 2013;79(4): 506–11. 10.4103/0378-6323.113080 23760320

[27] Yang Y, Xu J, Li F, Zhu X. Combination therapy of intravenous immunoglobulin and corticosteroid in the treatment of toxic epidermal necrolysis and Stevens-Johnson syndrome: a retrospective comparative study in China. Int J Dermatol. 2009;48(10): 1122–8. 10.1111/j.1365-4632.2009.04166.x 19775409

[28] Barron SJ, Del Vecchio MT, Aronoff SC. Intravenous immunoglobulin in the treatment of Stevens-Johnson syndrome and toxic epidermal necrolysis: a meta-analysis with meta-regression of observational studies. Int J Dermatol. 2015;54(1): 108–15. 10.1111/ijd.12423 24697283

[29] Roujeau JC, Bastuji-Garin S. Systematic review of treatments for Stevens-Johnson syndrome and toxic epidermal necrolysis using the SCORTEN score as a tool for evaluating mortality. Ther Adv Drug Saf. 2011;2(3): 87–94. 10.1177/2042098611404094 25083204 PMC4110817

[30] Lee HY, Dunant A, Sekula P, Mockenhaupt M, Wolkenstein P, Valeyrie-Allanore L, et al The role of prior corticosteroid use on the clinical course of Stevens-Johnson syndrome and toxic epidermal necrolysis: a case-control analysis of patients selected from the multinational EuroSCAR and RegiSCAR studies. Br J Dermatol. 2012;167(3): 555–62. 10.1111/j.1365-2133.2012.11074.x 22639874

[31] Stella M, Clemente A, Bollero D, Risso D, Dalmasso P. Toxic epidermal necrolysis (TEN) and Stevens-Johnson syndrome (SJS): experience with high-dose intravenous immunoglobulins and topical conservative approach. A retrospective analysis. Burns. 2007;33(4): 452–9. 10.1016/j.burns.2006.08.014 17475410

[32] Lalosevic J, Nikolic M, Gajic-Veljic M, Skiljevic D, Medenica L. Stevens-Johnson syndrome and toxic epidermal necrolysis: a 20-year single-center experience. Int J Dermatol. 2014. 10.1111/ijd.12702 25385069

[33] Egger M, Davey Smith G, Schneider M, Minder C. Bias in meta-analysis detected by a simple, graphical test. BMJ. 1997;315(7109): 629–34. 9310563 10.1136/bmj.315.7109.629 PMC2127453

[34] Higgins JP, Thompson SG. Quantifying heterogeneity in a meta-analysis. Stat Med. 2002;21(11): 1539–58. 10.1002/sim.1186 12111919

[35] DerSimonian R, Laird N. Meta-analysis in clinical trials. Control Clin Trials. 1986;7(3): 177–88. 3802833 10.1016/0197-2456(86)90046-2

[36] Zhang X. Analysis on immunoglobulin and corticosteroid combination therapy on pediatric Stevens-Johnson syndrome. World Health Digest. 2013;10(17): 69–70.[Article in Chinese]

[37] Jiang TF, Mi ZL, Chen YP, Ge WC. Clinical observations of the effects of high-dose immunoglobulin and steroid combination therapy on Stevens-Johnson syndrome. Zhejiang Clin Med. 2007;9(5): 634. [Article in Chinese]

[38] Wang M, Shang T, Huang YJ, Zhang YZ. Clinical analysis on toxic epidermal necrolysis treated by immunoglobulin and corticosteroid combination therapy. Chin J Lepr Skin Dis. 2010;26(7): 529. [Article in Chinese]

[39] Xia ZH. Clinical analysis on 35 cases with toxic epidermal necrolysis. Clin Educ Gen Pract. 2012;10(6): 670–1. [Article in Chinese]

[40] Zhu QY, Ma L, Luo XQ, Huang HY. Toxic epidermal necrolysis: performance of SCORTEN and the score-based comparison of the efficacy of corticosteroid therapy and intravenous immunoglobulin combined therapy in China. J Burn Care Res. 2012;33(6): e295–308. 10.1097/BCR.0b013e318254d2ec 22955159

[41] Dai Z, Pu XY, Li YQ. High dose immunoglobulin combined with methylprednisolone in the treatment of severe drug eruption clinical observation nursing. J Front Med. 2012;23: 30–1. [Article in Chinese]

[42] Jin LH, Wang YF. Clinical observations of the effects of immunoglobulin and steriod combination therapy on severe cutaneous adverse reactions. Chin Pract Med. 2011;6(3): 139–40. [Article in Chinese]

[43] Liu RR, Zhu H, He CD. Clinical analysis of corticosteroid and immunoglobulin combination therapy on severe adverse drug reactions. J Chin Pract Diagn Ther. 2012;26(7): 698–9. [Article in Chinese]

[44] Lu WS, Hu B, Liu JL, Zhao ZL, Wu AL, Zhang SP, et al Clinical analysis and treatment of Stevens-Johnson syndrome and toxic epidermal necrolysis. Chin J Clin Healthc. 2013;16(2): 133–5. [Article in Chinese]

[45] Brown KM, Silver GM, Halerz M, Walaszek P, Sandroni A, Gamelli RL. Toxic epidermal necrolysis: does immunoglobulin make a difference? J Burn Care Rehabil. 2004;25(1): 81–8. 10.1097/01.BCR.0000105096.93526.27 14726744

[46] Campione E, Marulli GC, Carrozzo AM, Chimenti MS, Costanzo A, Bianchi L. High-dose intravenous immunoglobulin for severe drug reactions: efficacy in toxic epidermal necrolysis. Acta Derm Venereol. 2003;83(6): 430–2. 10.1080/00015550310005852 14690337

[47] Kim KJ, Lee DP, Suh HS, Lee MW, Choi JH, Moon KC, et al Toxic epidermal necrolysis: analysis of clinical course and SCORTEN-based comparison of mortality rate and treatment modalities in Korean patients. Acta Derm Venereol. 2005;85(6): 497–502. 10.1080/00015550510038232 16396796

[48] Lissia M, Figus A, Rubino C. Intravenous immunoglobulins and plasmapheresis combined treatment in patients with severe toxic epidermal necrolysis: preliminary report. Br J Plast Surg. 2005;58(4): 504–10. 10.1016/j.bjps.2004.12.007 15897036

[49] Rajaratnam R, Mann C, Balasubramaniam P, Marsden JR, Taibjee SM, Shah F, et al Toxic epidermal necrolysis: retrospective analysis of 21 consecutive cases managed at a tertiary centre. Clin Exp Dermatol. 2010;35(8): 853–62. 10.1111/j.1365-2230.2010.03826.x 20456393

[50] Trent JT, Kirsner RS, Romanelli P, Kerdel FA. Analysis of intravenous immunoglobulin for the treatment of toxic epidermal necrolysis using SCORTEN: The University of Miami Experience. Arch Dermatol. 2003;139(1): 39–43. 12533162 10.1001/archderm.139.1.39

[51] Bachot N, Revuz J, Roujeau JC. Intravenous immunoglobulin treatment for Stevens-Johnson syndrome and toxic epidermal necrolysis: a prospective noncomparative study showing no benefit on mortality or progression. Arch Dermatol. 2003;139(1): 33–6. 12533161 10.1001/archderm.139.1.33

[52] Teo L, Tay YK, Liu TT, Kwok C. Stevens-Johnson syndrome and toxic epidermal necrolysis: efficacy of intravenous immunoglobulin and a review of treatment options. Singapore Med J. 2009;50(1): 29–33. 19224081

[53] Yeung CK, Lam LK, Chan HH. The timing of intravenous immunoglobulin therapy in Stevens-Johnson syndrome and toxic epidermal necrolysis. Clin Exp Dermatol. 2005;30(5): 600–2. 10.1111/j.1365-2230.2005.01863.x 16045716

[54] Schwartz RA, McDonough PH, Lee BW. Toxic epidermal necrolysis: Part II. Prognosis, sequelae, diagnosis, differential diagnosis, prevention, and treatment. J Am Acad Dermatol. 2013;69(2): 187 e1-16; quiz 203–4. 10.1016/j.jaad.2013.05.002 23866879

[55] Yip VL, Alfirevic A, Pirmohamed M. Genetics of immune-mediated adverse drug reactions: a comprehensive and clinical review. Clin Rev Allergy Immunol. 2015;48(2–3): 165–75. 10.1007/s12016-014-8418-y 24777842

[56] Kim DH, Yoon KC, Seo KY, Lee HS, Yoon SC, Sotozono C, et al The role of systemic immunomodulatory treatment and prognostic factors on chronic ocular complications in Stevens-Johnson syndrome. Ophthalmology. 2015;122(2): 254–64. 10.1016/j.ophtha.2014.08.013 25262319

[57] Yip LW, Thong BY, Tan AW, Khin LW, Chng HH, Heng WJ. High-dose intravenous immunoglobulin in the treatment of toxic epidermal necrolysis: a study of ocular benefits. Eye (Lond). 2005;19(8): 846–53. 15389280 10.1038/sj.eye.6701653

[58] Kim KH, Park SW, Kim MK, Wee WR. Effect of age and early intervention with a systemic steroid, intravenous immunoglobulin or amniotic membrane transplantation on the ocular outcomes of patients with Stevens-Johnson syndrome. Korean J Ophthalmol. 2013;27(5): 331–40. 10.3341/kjo.2013.27.5.331 24082770 PMC3782578

[59] de Prost N, Mekontso-Dessap A, Valeyrie-Allanore L, Van Nhieu JT, Duong TA, Chosidow O, et al Acute respiratory failure in patients with toxic epidermal necrolysis: clinical features and factors associated with mechanical ventilation. Crit Care Med. 2014;42(1): 118–28. 10.1097/CCM.0b013e31829eb94f 23989174

[60] Duong TA, de Prost N, Ingen-Housz-Oro S, Carrie AS, Zerah F, Valeyrie-Allanore L, et al Stevens-Johnson syndrome and toxic epidermal necrolysis: follow-up of pulmonary function after remission. Br J Dermatol. 2015;172(2): 400–5. 10.1111/bjd.13505 25496398

[61] Woo T, Saito H, Yamakawa Y, Komatsu S, Onuma S, Okudela K, et al Severe obliterative bronchitis associated with Stevens-Johnson syndrome. Intern Med. 2011;50(22): 2823–7. 22082897 10.2169/internalmedicine.50.5582

[62] Sekula P, Dunant A, Mockenhaupt M, Naldi L, Bouwes Bavinck JN, Halevy S, et al Comprehensive survival analysis of a cohort of patients with Stevens-Johnson syndrome and toxic epidermal necrolysis. J Invest Dermatol. 2013;133(5): 1197–204. 10.1038/jid.2012.510 23389396

[63] Alejandria MM, Lansang MA, Dans LF, Mantaring JB, 3rd. Intravenous immunoglobulin for treating sepsis, severe sepsis and septic shock. Cochrane Database Syst Rev. 2013;9: CD001090. 10.1002/14651858.CD001090.pub2 PMC6516813 24043371

[64] Kim JD, Choi DL, Han YS. Fourteen successful consecutive cases of ABO-incompatible living donor liver transplantation: new simplified intravenous immunoglobulin protocol without local infusion therapy. Transplant Proc. 2014;46(3): 754–7. 10.1016/j.transproceed.2013.11.100 24767341

[65] Creamer D, Walsh SA, Dziewulski P, Exon LS. Guidelines for the management of Stevens-Johonson syndrome/toxic epidermal necrolysis in adults 2016. Br J Dermatol. 2016;174: 11954–227. 10.1111/bjd.14530 27317286

