# Supplementary material for: The Effect of Intravenous Immunoglobulin Combined with Corticosteroid on the Progression of Stevens-Johnson Syndrome and Toxic Epidermal Necrolysis: A Meta-Analysis
Source: PLoS One. 2016 Nov 30;11(11):e0167120. doi: 10.1371/journal.pone.0167120 (PMC5130247; doi:10.1371/journal.pone.0167120)
Supplement: S1 File — (DOC) [file pone.0167120.s001.doc]

("Stevens-Johnson Syndrome" OR "SJS" OR "Toxic Epidermal Necrolysis" OR "TEN") AND ("IVIG" OR "intravenous immunoglobulin")
